# Impact of Social Determinants of Health on Women’s Satisfaction with Their Sexual Life and Its Relationship with the Use of Psychotropic Drugs: A Cross-Sectional Study

**DOI:** 10.3390/jcm11092320

**Published:** 2022-04-21

**Authors:** Regina Ruiz de Viñaspre-Hernández, Iván Santolalla-Arnedo, Rosana Garrido-Santamaría, Michał Czapla, Clara Isabel Tejada-Garrido, Juan Luis Sánchez-González, Esther Sapiña-Beltrán, Verónica Iriarte-Moreda, María Estela Colado-Tello, Vicente Gea-Caballero, Raúl Juárez-Vela

**Affiliations:** 1 Research Group in Care GRUPAC, Department of Nursing, University of La Rioja, 26004 Logroño, Spain; reruizde@unirioja.es (R.R.d.V.-H.); rosana.garrido@unirioja.es (R.G.-S.); michal.czapla@umw.edu.pl (M.C.); ester.sapina@unirioja.es (E.S.-B.); veronica.iriarte@unirioja.es (V.I.-M.); maria-estela.colado@unirioja.es (M.E.C.-T.); raul.juarez@unirioja.es (R.J.-V.); 2 Rioja Health Service SERIS, Government of La Rioja, 26004 Logroño, Spain; 3 Laboratory for Experimental Medicine and Innovative Technologies, Department of Emergency Medical Service, Wroclaw Medical University, 51-618 Wroclaw, Poland; 4 Department of Nursing and Physiotherapy, University of Salamanca, 37008 Salamanca, Spain; juanluissanchez@usal.es; 5 Faculty of Health Sciences, International University of Valencia, 46002 Valencia, Spain; vagea@universidadviu.com

**Keywords:** orgasm, health behavior, psychotropic drugs, social determinants of health

## Abstract

Sexual satisfaction (SS) is defined as an affective response arising from one’s subjective evaluation of the positive and negative dimensions associated with one’s sexual relationship. It is an important indicator of health. In women, SS has an important personal component consisting of the physical experiences of pleasure and the positive feelings and emotions that they experience in their affective-sexual relationships. The socioeconomic position is determined by income, educational level, and work, and it conditions women’s sexual health. We aimed to assess whether social determinants of health (income, education, work, and gender) are associated with women’s sexual satisfaction and to identify whether the impact of social determinants on sexual satisfaction differs with psychotropic consumption. We conducted a cross-sectional study designed to assess the association between variables related to the social determinants of health (work, education, income, and gender) and sexual satisfaction in women of reproductive age in La Rioja (Spain). The women in this study ranged in age from 17 to 52 years, with a mean age of 33.4 (Standar Deviation 8.6). Most were Spanish (82.9%), had undertaken non-compulsory specialized education (84%), and worked (72.7%). Regarding their relationships, 87% maintained monogamous relationships, 84.5% had stable relationships, and 65.7% lived with their partners. In total, 12.3% of the women were taking psychotropic drugs prescribed for the treatment of anxiety and/or depression. We observed that SS is significantly lower among women who have only undertaken compulsory education (Student-*t* = −4.745; *p* < 0.01), in those who have unstable affective-sexual relationships (Student-*t* = −2.553; *p* < 0.01), and in those who take psychotropic drugs (Student-*t* = −4.180, *p* < 0.01). We conclude that the social determinants of health such as education, not continuing to study beyond compulsory education, gender, and taking psychoactive drugs have a significant impact on women’s degree of satisfaction with their sexual life.

## 1. Introduction

Sexual satisfaction (SS), defined by [1] as “an affective response arising from one’s subjective evaluation of the positive and negative dimensions associated with one’s sexual relationship” (p. 268), is an important indicator of health [2] and provides physical and emotional well-being, greater satisfaction with established affective relationships, better quality of life, and, in general, greater happiness [3,4].

SS is a complex concept with physical, emotional, relational, and cultural dimensions. Multiple factors influence the SS of women, and their impact differs widely depending on the socioeconomic and cultural context in which they are studied [5,6]. Sexual satisfaction is often opposed to sexual dysfunction; however, there are women who do not feel satisfied with their sexual life without having any sexual dysfunction [7,8]. In women, SS has an important personal component consisting of the physical experiences of pleasure and the positive feelings and emotions that they experience in their affective-sexual relationships [9]. However, sexual experiences are not always suited to their tastes, needs, and expectations, nor are they freely chosen [9,10,11]. Social determinants of health (SDH—income, education, work, and gender), that is, the socioeconomic structure in which women live, can favor or hinder the ability to negotiate and decide how, when, and with whom to establish a sexual exchange [12]. The socioeconomic position is determined by income, educational level, and work, and it conditions women’s sexual health [13], although it does so indirectly, through intermediate determinants such as access to services, health resources, material goods, lifestyles, and health behaviors, psychosocial and biological factors [14,15]. Regarding the gender role assigned and assumed by women, it has been observed that how women build their affective-sexual relationships and the structure of their families is mediated by the predominant social discourses [16].

A factor that significantly interferes with sexual life and is strongly associated with the female gender is the use of psychotropic drugs [17]. Psychotropic agents can affect all phases of the sexual response due to their interaction with serotonin and dopamine [18]. Spain is one of the Western European countries with the highest consumption of psychotropic drugs, mainly anxiolytics, antidepressants, and hypnotics [19]. Consumption rates in women are double or even triple those of men in all age groups, women experience a more rapid progression towards addiction than men [20], and most prescriptions are chronic or long-term [21]. Research carried out from a gender perspective shows the influence of gender stereotypes on prescription and consumption [22,23].

When the social determinants of health (work, education, income, and gender) are unfavorable for a human group, their risk of suffering from health problems can negatively affect their sexual life increases, causing situations of vulnerability and inequity. The analysis of SS with the DSS approach has been little explored, despite being an important reference framework in the study of community health and the definition of public health policies [13,24]. We believe that in Spain, social determinants could explain the greater difficulty faced by some groups of women in establishing egalitarian and positive relationships that result in their sexual satisfaction.

This study aimed to assess whether social determinants of health (income, education, work, and gender) are associated with women’s sexual satisfaction and to identify whether the impact of social determinants on sexual satisfaction differs with psychotropic consumption.

## 2. Materials and Methods

We conducted a cross-sectional study designed to assess the association between variables related to the social determinants of health (work, education, income, and gender) and sexual satisfaction in women of reproductive age in La Rioja (Spain). The data for this study were collected through a questionnaire developed ad hoc to document the sociodemographic, cultural, family, and health profiles of the women who participated in the study. It was a cultural adaptation and validation into Spanish of the sexual satisfaction scale (SSS-W-E) [25]. This study showed the reliability (Cronbach’s α = 0.93) and construct validity of the questionnaire, and of its scales, to measure sexual satisfaction in this population. Sexually active women aged ≥16 who attended the family planning clinic from June 2020 to February 2021 were recruited consecutively. We excluded women who did not speak Spanish, those who could not complete the scale due to mental or other disorders, and those who did not give their consent to participate in this study. In total, 316 women signed the consent form and were enrolled in the study. The data were collected by three midwives who were trained for this purpose. The outcome variable sexual satisfaction was defined as overall contentment with emotional and sexual aspects of the sex life (contentment). This is the first of the five domains (contentment, communication, compatibility, concern about the relationship, and personal concerns) that are assessed with the questionnaire (SSS-W-E). The scale that measures contentment is made up of 6 items: (1) I feel content with my present sex life; (2) I feel something is missing from my present sex life; (3) I feel I do not have enough emotional closeness; (4) I feel content with the frequency of sexual intimacy; (5) I do not have any problems or concerns about sex; (6) Overall, I am satisfied with my sex life. Women are instructed to rate their level of agreement with each item using a Likert scale from 1 = strongly disagree to 5 = strongly agree. The score range for each domain is 6–30, and it is calculated by adding the scores of the individual items comprising each separate dimension [25,26]. The “contentment” domain correlated significantly with the rest of the domains that make up the questionnaire (communication: r = 0.536, *p* < 0.01; compatibility: r = 0.605, *p* < 0.01; concern about the relationship: r = 0.506, *p* < 0.01; and personal concerns: r = 0.583, *p* < 0.01). Predictor variables were age (years); nationality or country of origin: Spain or other; level of education: compulsory education (basic education ends at 16 years old) or non-compulsory education (specialized education after basic education); annual income: less than EUR 12,000, from EUR 12,001 to EUR 20,000, from EUR 20,001 to EUR 35,000, from EUR 35,001 to EUR 60,000, or more than EUR 60,000; employment situation: unemployed, salaried employee or self-employed; affective-sexual relationship: monogamous (with one partner) or non-monogamous (without a partner or with two or more partners at the same time); stability of the relationship: stable or unstable; cohabiting with the partner: yes or no; number of children; psychotropic drug use (women using psychotropic drugs for over three months): yes or no. The variables were summarized using means and standard deviations (SD), frequencies, and percentages, depending on the type of variable. In the bivariate analysis, it was measured whether there were significant differences (*p* < 0.05) in the sexual satisfaction (contentment) of the women grouped in the categories of the qualitative predictor variables. Depending on the type of distribution, we used the t-Student and ANOVA or the Mann–Whitney U and Kruskall–Wallis test and their post hoc tests if necessary. When the predictor variable was quantitative, its association with the outcome variable was estimated using Pearson’s r coefficient. Following the central limit theorem, we considered that the samples of the various categories with a size ≥30 women would approximately follow a normal distribution, and therefore, parametric tests could be used to measure the differences [27,28].

We used multivariate linear analysis to identify the predictor variables involved in the outcome of sexual satisfaction. The variables that show a value of *p* ≤ 0.2 in the bivariate analysis were included [29]. Those variables that maintained their statistical significance (*p* < 0.05) were left in the final model. We performed all statistical analyses using SPSS Software version 23 (IBM Corporation, New Orchard Road Armonk, New York, NY, USA). The study was approved by the ethics committee of the Rioja Biomedical Research Center (CIBIR) (reference CEImLar P.I. 386).

## 3. Results

### 3.1. Baseline Characteristics

The women in this study ranged in age from 17 to 52 years, with a mean age of 33.4 (SD 8.6). Most were Spanish (82.9%), had undertaken non-compulsory specialized education (84%), and worked (72.7%). Regarding their relationships, 87% maintained monogamous relationships, 84.5% had stable relationships, and 65.7% lived with their partners. In total, 12.3% of the women were taking psychotropic drugs prescribed for the treatment of anxiety and/or depression. The mean sexual satisfaction score was 20.3 (SD 5.9) with a range of 6–30.

### 3.2. Changes between the Study Groups

When analyzing the differences in the sociodemographic and family structure profile between the group of women who take psychotropic drugs (USD) and the group of women who do not (NUSD), we find that: in the first group (USD), the mean age is higher, although this difference does not reach the limit of statistical significance (35.8 years vs. 33.03, *p* = 0.06); a higher percentage of women have only undertaken compulsory education (30.8% vs. 13.4%, *p* < 0.01), are unemployed (48.7 vs. 32.3%, *p* = 0.06), and have unstable affective relationships (28.9% vs. 13.7%, *p* = 0.02) compared to the second group (NUSD) (Table 1).

### 3.3. Predictors of Sexual Satisfaction

In the bivariate analysis that includes all the women studied (N = 316), we observed that SS is significantly lower among women who have only undertaken compulsory education (Student-*t* = −4.745; *p* < 0.01), in those who have unstable affective-sexual (Student-*t* = −2.553; *p* < 0.01), and in those who take psychotropic drugs (Student-*t* = −4.180, *p* < 0.01). There were also significant differences in SS depending on employment status (ANOVA, F = 7.554, gl = 2, *p* <0.01); however, when estimating the differences by pairs using the Bonferroni correction, we found that self-employed women were significantly more satisfied with their sexual life than unemployed women (*p* = 0.05) and then those with salaried work (*p* < 0.01), but the difference was not significant between unemployed women and women with salaried work. Finally, there was a small but significant negative correlation between the number of children and SS (*r* = −0.147, *p* < 0.01) (Table 2).

If we observe the associations found when disaggregating the data by psychotropic drug use, we see that SS maintains its association with the education variable in both groups (*p* = 0.01 and *p* < 0.01, respectively). In the first group (USD), a positive and statistically significant correlation was also found between age and sexual satisfaction (*r* = 0.477, *p* < 0.01). In the second group (NUSD), sexual satisfaction did not show a significant association with age but did with the number of children (*r* = −0.166, *p* < 0.01) (Table 2). 

The multivariate model that includes all women shows that the educational level (compulsory vs. non-compulsory education) and the use or not of psychotropic drugs explain 10.3% of the variance in the value of sexual satisfaction. Women who only attended compulsory education had almost 4 points less sexual satisfaction than women with higher education, and women who consume psychotropic drugs had around 3.5 points less sexual satisfaction than those who do not require psychotropic drug

When we control for the effect of drug use by studying the two groups of women (USD and NUSD) independently, we observe that the variables education and age that make up the model of the USD group explain 31.6% of the variance, while in the NUSD group, the variables education and number of children only explain 5.8%. In both groups, not continuing to study beyond compulsory education decreases SS by around 3 points. In the first group (USD), for each year older, satisfaction increased by 0.2 points, and in the second group (NUSD), for each child, satisfaction decreased 0.6 points (Table 3).

## 4. Discussion

In this study, a higher educational level is an independent protective factor for SS. A previous study with Spanish women corroborates this result [13], as do studies with women from other countries [30] and with different health problems: vulvodynia [31], multiple sclerosis [32], or infertility [6]. However, not all studies show this association [33,34,35]. In this study, when we controlled for the effect of psychotropic drugs, education was shown to be the social determinant most strongly associated with SS. Women with a higher educational level have more opportunities to lead a healthy life and seek advice and help more effectively to solve their health problems [36,37]. Better physical and mental health predicts higher sexual satisfaction [3]. In addition, the beneficial effect of a higher educational level on health seems to be stronger in women than in men [38]. On the other hand, the dominant meritocratic discourse in Western society leads people to perceive education as a personal achievement, a tool to break down the boundaries between social classes and make them permeable. It has been observed that, in high-income countries, education plays an important role in the structure of the self-concept that people develop, above other identity concepts such as ethnicity, race, or gender [39]. People form meaningful identities based on their level of education and hold stereotypes about other people on this basis [40]. At a higher educational level, education acquires greater identity weight and influences aspects such as self-esteem [41], confidence [42,43], or the choice of partner [42], which are protective factors for sexual satisfaction [30]. In Spain, women with a lower educational level are more likely to have unwanted [13] or unsafe [24] sexual relations.

In our study, women’s economic level does not seem to influence their SS. We did find a higher degree of sexual satisfaction in the group of self-employed women compared to those who work for other people, probably because these self-employed women (freelancers or businesswomen) more often had undertaken university education [44], which would explain why in the multivariate model the employment relationship ceased to be significant, and the significance of education was maintained. Access to the Spanish national health system is free and universal, so the effect of the economic level, the origin of the people, or the work they do may have a lesser impact on health outcomes at the community level than in other countries. The personal and social tools provided by education result in better physical, mental, and sexual health, with the final result of greater sexual satisfaction and less use of psychotropic drugs, as shown by our data and collected in the literature [37,45].

The psychotropic drugs were prescribed for the treatment of anxiety and/or depression, and their consumption is related to worse values of sexual satisfaction, which is consistent with the literature [18,46,47,48]. Results show such women had a statistically significantly higher proportion of unemployed and in unstable affective relationships. This would strongly suggest that these women were more stressed. It is also noteworthy that their educational level is significantly lower than that of the group of women who do not take psychotropic drugs. These data corroborate the data provided by other studies in which it is evident that women with this same profile are more likely to be chronic users of psychotropic drugs [21,45]. Poor mental health itself, which shows a significantly higher prevalence in women with less education [49,50], correlates with lower sexual satisfaction [51]. This relationship feeds back: it has been observed that women with lower levels of sexual satisfaction have a higher risk of suffering from depression and/or anxiety throughout their lives and women with symptoms of anxiety and depression have a higher risk of sexual dissatisfaction [52]. On the other hand, the drugs that are administered for anxiety and depression have a significant deleterious effect on sexuality. Chronic use of antidepressants seems to alter the sensitivity and number of nerve receptors on which they act, giving rise to a chronic mood disorder in people who maintain their use for years [21]. In Spain, the phenomenon of the medicalization of mental health is worrying, on the rise, and mainly affects women [22]. Among the other gender-sensitive variables included in this study, age is not associated with SS except in the group of women taking psychotropic drugs. In the literature, the association between age and SS is controversial [35]; we found that for women using psychotropic drugs, being older is correlated with greater satisfaction with their sexual life. Younger women with younger, more sexually active partners are more likely to engage in sexual intercourse to avoid disappointing their partners than older women, leading to dissatisfaction and unhappiness [53]. Several studies show that women’s sexual satisfaction depends on the satisfaction of their partners to a greater extent than that of men [54]. On the other hand, in clinical practice, it has been observed that some women undergoing psychotropic treatment, over time, may experience an improvement, at least partial, in their sexual dysfunction [46]. The negative impact of the number of children on women’s sexual satisfaction could be a consequence of the fact that childcare, which in Spain occupies women to a greater extent than men [55], reduces the amount of time available for interactions and their frequency, and both aspects have been closely associated with sexual satisfaction [3,56]. On the other hand, there is usually a greater number of children in relationships of longer duration, which has been associated with a loss of sexual interest in the couple that affects the sexual satisfaction of both [57,58]. Other gender-sensitive variables, such as the type of relationship established or living with a partner, are not associated with satisfaction. Women who maintained a stable relationship had higher levels of satisfaction, but when adjusting the variable for education and the use of psychotropic drugs, the difference in satisfaction between them was no longer significant. Other studies did find greater satisfaction when the woman has a stable relationship [59,60,61].

## 5. Limitations

This study has limitations; first of all, the data come from another study whose main objective was to adapt and validate the sexual satisfaction questionnaire for women (SSS-W) [26] in Spanish (SSS-W-E) [25]. Although the analysis of predictive factors of SS in women was a secondary objective of the other study, the sample was estimated based on the main objective. This study has limitations; first of all, the data come from another study whose main objective was to adapt and validate the sexual satisfaction questionnaire for women (SSS-W) [26] in Spanish (SSS-W-E) [25]. Although the analysis of predictive factors of SS in women was a secondary objective of the other study, the sample was estimated based on the main objective. This study did not include measures of anxiety or depression, so cannot say whether it is anxiety/depression or psychotropic drug use (and/or stress) that was associated with lower SS and, in no case, data from this study provide evidence of causality, only of association. On the other hand, the place where the sample was collected, a family planning center, is likely to have conditioned the characteristics of the women studied in such a way that the percentage of lesbians may be lower than that of heterosexual or bisexual women. In addition, the data collection for this study was carried out during the CO-VID-19 pandemic, we do not know to what extent the care restrictions could affect the type of population that attended the health service. Lastly, although the percentage of women included in the psychotropic drug use group corresponds to the percentage of women with psychological morbidity, mostly anxiety and depression, diagnosed in the community in 2017 and the use of psychotropic drugs in Spain [62], the sample size achieved is small, which reduces the predictive capacity of the multivariate model developed for this group of women.

## 6. Implications for Practice

Policy initiatives that combat school dropout and provide options to improve women’s specialized academic training could be effective in improving women’s sexual satisfaction. At a clinical level, exploring women’s SS and educational level could help identify situations of vulnerability (lack of knowledge of current legislation on the right to sexual and reproductive health, lack of resources for negotiating relationships, or low self-esteem) in which we can intervene. Concerning women who use psychotropic drugs, public health initiatives should provide therapeutic options other than pharmacological treatment in response to the demand for their use in psychological distress in women, integrate the gender perspective in the prescription of psychoactive drugs, and discuss with women the repercussions they have on their SS. An important challenge for future research would be to establish the intermediate mechanisms through which low educational level results in both the use of psychoactive drugs and sexual satisfaction in Spanish women.

## 7. Conclusions

In this study, we found data that show that two situations related to the social determinants of health: education, not continuing to study beyond compulsory education and gender, and taking psychoactive drugs have a significant impact on women’s degree of satisfaction with their sexual life.

## Figures and Tables

**Table 1:** Baseline characteristics and changes between the study groups.

Variables	All Women (N = 316)	USD Group (N = 39)	NUSD Group (N = 277)	
	N (%) or Mean (SD)	N (%) or Mean (SD)	N (%) or Mean (SD)	*p*
Age	33.36 (8.58)	35.77 (9.42)	33.03 (8.42)	0.06
Country				
Spain	262 (82,9%)	31 (79.5%)	231 (83.4%)	0.54
Other countries	54 (17.1%)	8 (20.5%)	46 (16.6%)	
Education				
Compulsory	49 (15.5%)	12 (30.8%)	37 (13.4%)	<0.01
Non-compulsory	267 (84.5%)	27 (69.2%)	240 (86.6%)	
Income (EUR/year)				
<12.000	73 (23.1%)	10 (25.6%)	63 (22.7%)	0.33
12.00–20.00	98 (31.0%)	9 (23.1%)	89 (32.2%)	
20.000–35.000	79 (25.0%)	8 (20.5%)	71 (25.6%)	
35.000–60.000	48 (15.2%)	10 (25.6%)	38 (13.7%)	
>60.000	18 (5.7%)	2 (5.0%)	16 (5.8%)	
Employment situation				
Unemployed	102 (32.3%)	19 (48.7%)	83 (30.0%)	0.06
Salaried employee	175 (55.4%)	17 (43.6%)	158 (57.0%)	
Self-employed	39 (12.3%)	3 (7.7%)	36 (13.0%)	
Sexual Relationship				
Monogamous	275 (87.0%)	34 (87.2%)	241 (87.0%)	0.98
Non-monogamous	41 (13.0%)	5 (12.8%)	36 (13.0%)	
Stability of the relationship				
Stable	261 (84.5%)	27 (71.1%)	234 (86.3%)	0.02
Unstable	48 (15.5%)	11 (28.9%)	37 (13.7%)	
Cohabitation as a couple				
Yes	203 (65.7%)	27 (71.0%)	176 (63.5%)	0.46
No	106 (34.3%)	11 (29.0%)	95 (36.5%)	
Children	0.95 (1.14)	1.13 (1.10)	0.95 (1.14)	0.35
Psychotropic drug use				
No	277 (87.7%)			
Yes	39 (12.3%)			

Note: SD—standard deviation; USD—use of psychotropic drug; NUSD—no use of psychotropic drug.

**Table 2:** Association between women’s sexual satisfaction and variables related to social determinants of health.

Variables	Sexual Satisfaction					
	All Women		USD Group		NUSD Group	
	Mean (SD)	*p*	MEAN (SD)	*p*	Mean (SD)	*p*
Age	20.36 (5.94)	0.43	16.77	<0.01	20.81 (5.78)	0.20
Country						
Spain	20.35 (5.87)	0.77	17.10 (4.47)	0.38	20.79 (5.90)	0.91
Other countries	20.09 (5.45)		15.50 (4.78)		20.89 (5.20)	
Education						
Compulsory	16.82(4.81)	<0.01	14.17 (2.88)	0.01	17.68 (5.02)	<0.01
Non-compulsory	20.95 (5.73)		19.93 (4.66)		21.29 (5.75)	
Income (EUR/year)						
<12.000	19.33 (6.04)	0.33	16.70 (5.66)	0.60	19.75 (6.03)	0.34
12.00–20.00	20.30 (5.62)		16.11 (3.62)		20.72 (5.63)	
20.000–35.000	20.87 (5.33)		18.75 (4.77)		21.11 (5.36)	
35.000–60.000	20.21 (6.42)		16.40 (4.32)		21.21 (6.54)	
>60.000	22.11 (5.79)		14 (1.41)		23.13 (5.29)	
Employment situation						
Unemployed	20.71 (6.26)	<0.01	16.16 (5.19)	0.55	21.75 (6.00)	0.59
Salaried employee	19.42 (5.42)		17.53 (3.86)		19.63 (5.53)	
Self-employed	23.23 (5.27)		16.33 (4.16)		23.81 (4.97)	
Sexual Relationship						
Monogamous	20.46 (6.09)	0.48	17.80 (5.35)	0.59	20.93 (5.93)	0.35
Non-monogamous	19.71 (4.81)		16.62 (4.45)		19.97 (4.71)	
Stability of the relationship						
Stable	20.7 (5.8)	0.01	17.48 (4.42)	0.10	21.09	0.11
Unstable	18.4 (5.5)		15.00 (4.67)		19.41	
Cohabitation as a couple						
Yes	20.09 (5.87)	0.499	16.11 (6.01)	0.31	20.70 (5.86)	0.40
No	20.87 (5.77)		18.36(6.63)		21.16 (5.79)	
Children	20.36 (5.94)	0.01	16.77	0.90	20.81 (5.78)	0.01
Psychotropic drug use						
No	20.81 (5.78)	<0.01				
Yes	16.77 (4.52)					

Note: SD—standard deviation; USD—use of psychotropic drug; NUSD—no use of psychotropic drug.

**Table 3:** Prediction models of sexual satisfaction in women.

	All Women (N = 316)			USD Group (N = 39)			NUSD Group (N = 277)		
	B Coefficient	CI 95%	*p*	B Coefficient	CI 95%	*p*	B Coefficient	CI 95%	*p*
Constant	13.606	10.248–16.963	<0.01	4.689	(−1.472)–10.849	0.131	15.627	11.133–19.493	<0.01
Education	3.877	2.118–5.637	<0.01	2.941	0.183–5.699	0.037	3.075	1.041–5.109	<0.01
Age (Years)				0.199	0.062–0.335	<0.01			
Children (Number)							−0.593	(−1.200–0.013)	0.055
Psychotropic drug use	3.475	1.568–5.382	<0.01						
R^2^	0.103			0.316			0.058		

Abbreviations: USD—use of psychotropic drug; NUSD—no use of psychotropic drug; IC—confidence interval; R^2^—R-squared indicates the percentage of the dependent variable that the independent variables explain collectively.

## Data Availability

Data are available contacting with the corresponding author.
